# Biodistribution and radiation dosimetry of the positron emission tomography probe for AMPA receptor, [^11^C]K-2, in healthy human subjects

**DOI:** 10.1038/s41598-021-81002-3

**Published:** 2021-01-15

**Authors:** Mai Hatano, Tomoyuki Miyazaki, Yoshinobu Ishiwata, Waki Nakajima, Tetsu Arisawa, Yoko Kuroki, Ayako Kobayashi, Yuuki Takada, Matsuyoshi Ogawa, Kazunori Kawamura, Ming-Rong Zhang, Makoto Higuchi, Masataka Taguri, Yasuyuki Kimura, Takuya Takahashi

**Affiliations:** 1 Department of Physiology, Yokohama City University Graduate School of Medicine, Yokohama, Japan; 2 Department of Radiology, Yokohama City University Graduate School of Medicine, Yokohama, Japan; 3 Department of Anesthesiology, Yokohama City University Graduate School of Medicine, Yokohama, Japan; 4 Department of Radiopharmaceuticals Development, National Institute of Radiological Sciences, National Institutes for Quantum and Radiological Science and Technology, Chiba, Japan; 5 Department of Functional Brain Imaging Research, National Institute of Radiological Sciences, National Institutes for Quantum and Radiological Science and Technology, Chiba, Japan; 6 School of Data Science, Yokohama City University, Yokohama, Japan; 7 Department of Clinical and Experimental Neuroimaging, Center for Development of Advanced Medicine for Dementia, National Center for Geriatrics and Gerontology, Obu, Japan

**Keywords:** Ion channels in the nervous system, Diagnostic markers

## Abstract

[^11^C]K-2, a radiotracer exhibiting high affinity and selectivity for α-amino-3-hydroxy-5-methyl-4-isoxazole propionic acid receptors (AMPARs), is suitable for the quantification of AMPARs in living human brains and potentially useful in the identification of epileptogenic foci in patients. This study aimed to estimate the radiation doses of [^11^C]K-2 in various organs and calculate the effective dose after injection of [^11^C]K-2 in healthy human subjects. Twelve healthy male subjects were registered and divided into two groups (370 or 555 MBq of [^11^C]K-2), followed by 2 h whole-body scans. We estimated the radiation dose of each organ and then calculated the effective dose for each subject. The highest uptake of [^11^C]K-2 was observed in the liver, while the brain also showed relatively high uptake. The urinary bladder exhibited the highest radiation dose. The kidneys and liver also showed high radiation doses after [^11^C]K-2 injections. The effective dose of [^11^C]K-2 ranged from 5.0 to 5.2 μSv/MBq. Our findings suggest that [^11^C]K-2 is safe in terms of the radiation dose and adverse effects. The injection of 370–555 MBq (10 to 15 mCi) for PET studies using this radiotracer is applicable in healthy human subjects and enables serial PET scans in a single subject.

## Introduction

The glutamate α-amino-3-hydroxy-5-methyl-4-isoxazole propionic acid receptor (AMPAR) is widely distributed in the central nervous system and plays fundamental roles in neuronal function^1–7^. Furthermore, previous animal studies have suggested that AMPAR dysfunction underlies neuropsychiatric disorders^8–10^. These findings suggest that AMPARs may be a promising therapeutic target for these diseases. However, the development of drugs acting on AMPARs has been limited^11–13^. One major reason for this limitation is that we are unable to quantify the density of AMPARs in the brains of patients with neuropsychiatric disorders and to target appropriate diseases and patients in whom AMPAR-acting drug could exhibit clinical efficacy.

Recently, we developed a radioligand [^11^C]K-2 for AMPARs. Preclinical and clinical scans with positron emission tomography (PET) exhibited a high affinity and selectivity of the binding of [^11^C]K-2 to AMPARs, and PET imaging with [^11^C]K-2 is suitable for the quantification of AMPARs in living human brains^14^. Quantitative analysis of [^11^C]K-2 using healthy subjects revealed that the standardized uptake value ratio (SUVR) using white matter as reference, obtained from a 20-min scan, can be a surrogate marker for the binding potential (*BP*_ND_: a quantitative index of receptor density in PET imaging), indicating clinical usefulness of [^11^C]K-2^15^. Furthermore, we observed increased [^11^C]K-2 uptake in the clinically identified epileptogenic foci of patients with epilepsy^14^. Thus, [^11^C]K-2 can be a promising radiotracer to investigate the molecular pathology of neuropsychiatric disorders such as epilepsy, Alzheimer's disease, depression, and schizophrenia^16–18^.

In this clinical trial, we estimated the radiation doses of [^11^C]K-2 in various organs and calculated an effective dose per injected activity. Twelve healthy subjects were injected intravenously with a low (370 MBq) or high dose (555 MBq) of [^11^C]K-2 and underwent time-activity measurements in each organ using whole-body PET to define the tissue concentration of the injected radiotracer. The absorbed radiation dose was then estimated and the effective dose was calculated based on medical internal radiation dosimetry using OLINDA radiation dose evaluation software.

## Results

### Radiation dosimetry in mice

Prior to radiation dosimetry in human, biodistribution after [^11^C]K-2 injection in ddY mice was characterized to expect biodistribution in human (Supplemental Table 1). The highest uptake of [^11^C]K-2 was observed in the liver 15 min after injection, with a maximum of 32.02%ID/g and sustained uptake. Supplemental Table 2 shows the residence time of each source organs in mice, which could be translated to expect absorbed radiation doses in human using PMOD software as listed in Supplemental Table 3. The liver was expected to be the organ showing the highest absorbed radiation doses, 23.8 μGy/MBq in male and 31.5 μGy/MBq in female. Small intestine, gallbladder wall and kidneys showed relatively high absorbed radiation doses. Effective doses in human were expected to be 3.7 and 4.6 μSv/MBq in male and female, respectively (Supplemental Table 3). During this mice study, no abnormal behaviors related to the injection of [^11^C]K-2 were observed.

### Biodistribution of each source organ in human

**Figure 1:**
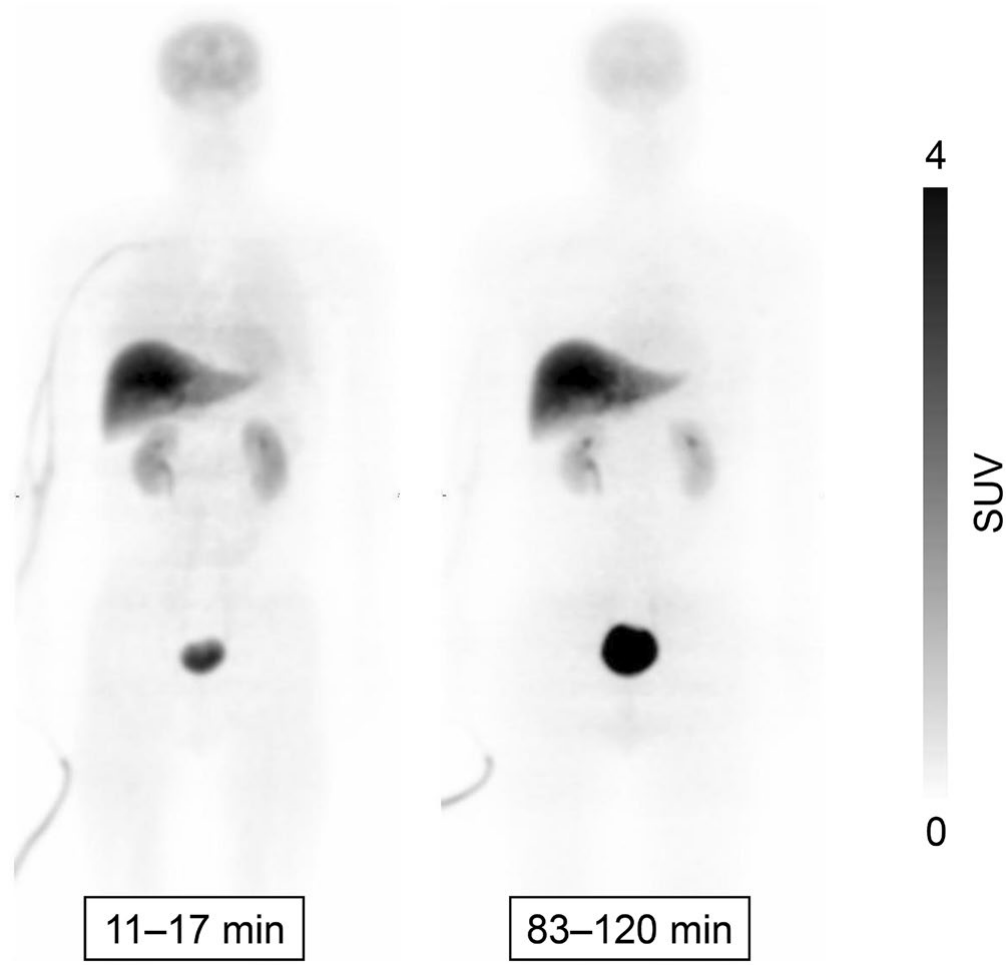
Whole-body PET images at different time points after injection of 366 MBq [^11^C]K-2. Whole-body decay-corrected PET images obtained at different times after injection of 366 MBq [^11^C]K-2 displayed using the same grayscale. Images were obtained at 11–17 and 83–120 min after intravenous injection of [^11^C]K-2 in a healthy male subject. All coronal slices were summed to create the images at different time points.

**Figure 2:**
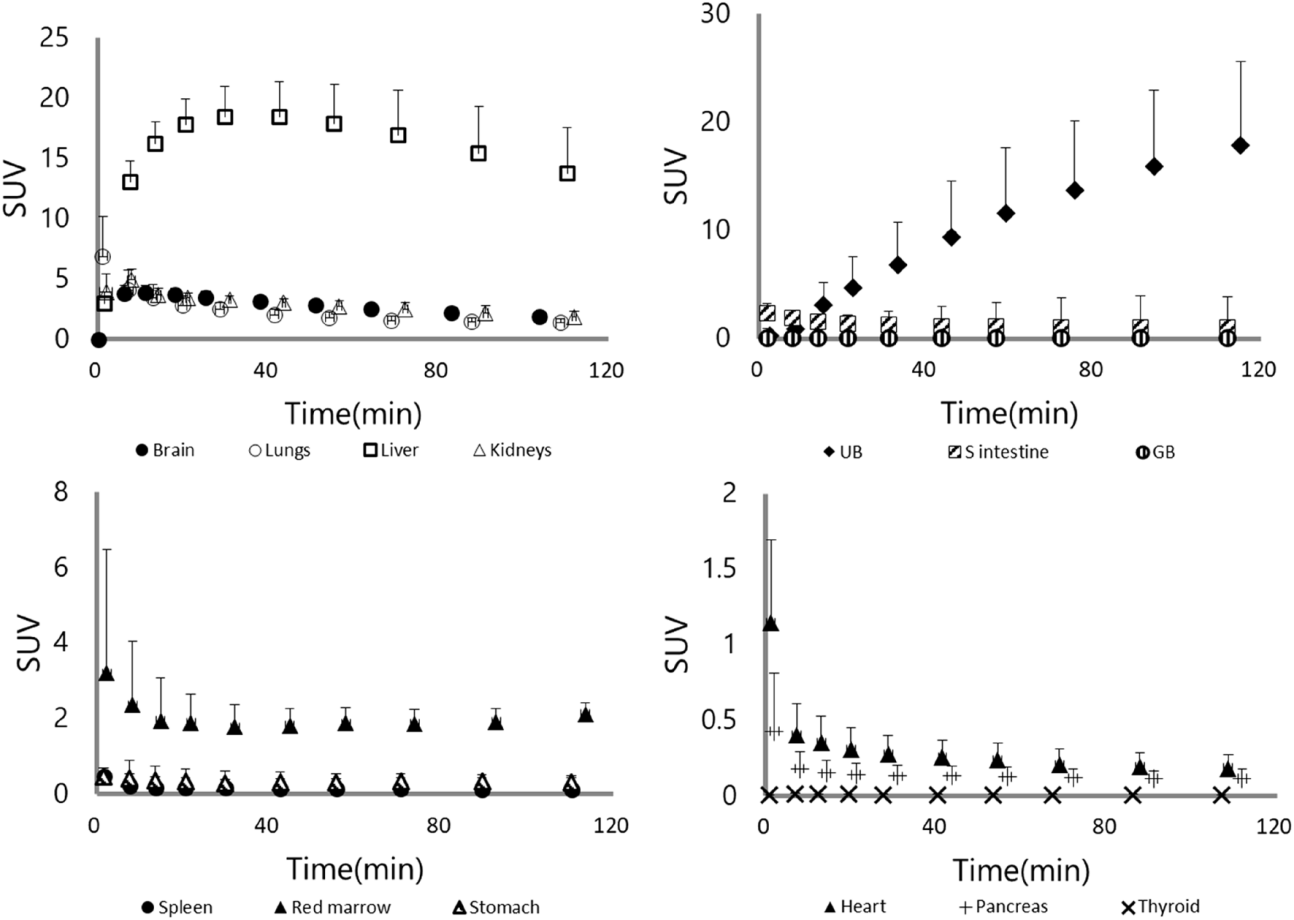
Time-activity curves for 13 organs after injection of 370 MBq [^11^C]K-2 in six healthy subjects. Decay-corrected time-activity curves for 13 organs after injection of 370 MBq [^11^C]K-2 in six healthy subjects. Symbols represent the mean radioactivity of each organ, expressed as SUV. Error bars reflect the standard deviation. UB, urinary bladder; S intestine, small intestine; GB, gallbladder.

**Figure 3:**
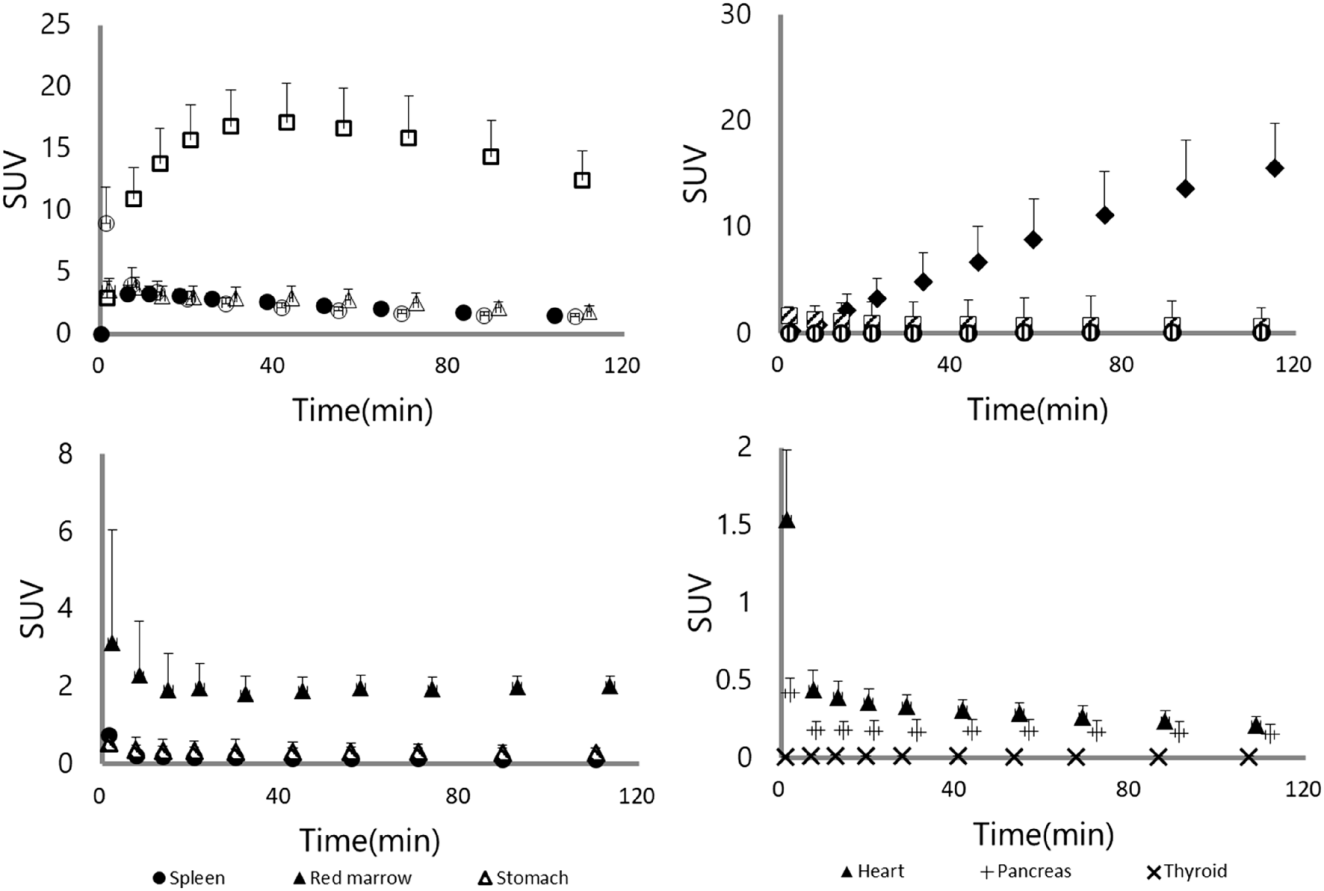
Time-activity curves for 13 organs after injection of 555 MBq [^11^C]K-2 in six healthy subjects. Decay-corrected time-activity curves for 13 organs after injection of 555 MBq [^11^C]K-2 in six healthy subjects. Symbols represent the mean radioactivity of each organ, expressed as SUV. Error bars reflect the standard deviation. UB, urinary bladder; S intestine, small intestine; GB, gallbladder.

To examine the biodistribution of [^11^C]K-2, 12 healthy subjects were recruited, divided into two groups, and injected [^11^C]K-2 at the dose of either 370 or 555 MBq (Table 1). We observed the visually identified uptake of [^11^C]K-2 in brain, lungs, heart, liver, spleen, stomach, kidneys, pancreas, gallbladder, small intestine, and urinary bladder on the PET images and treated the regions as source organs for radiation dosimetry calculation (Fig. 1). The highest uptake of [^11^C]K-2 was observed in the liver 30–42 min after injection, with a maximum of 18.4 and 17.1 in SUV after injection with 370 and 555 MBq of [^11^C]K-2, respectively, with subsequent slow clearance (Figs. 2 and 3). Urinary bladder was highest dose organ, showing sustained uptake with a maximum of 17.9 and 15.6 in SUV after injection with 370 and 555 MBq of [^11^C]K-2, respectively. Rapid uptake and washout were observed in following organs with a maximum SUV of 6.9 (370 MBq) and 8.9 (555 MBq) in the lungs, 1.1 (370 MBq) and 1.5 (555 MBq) in the heart, 0.48 (370 MBq) and 0.76 (555 MBq) in the spleen, 0.47 (370 MBq) and 0.55 (555 MBq) in the stomach, 0.42 (370 and 555 MBq) in the pancreas, 2.4 (370 MBq) and 1.6 (555 MBq) in the small intestine. The brain and kidneys showed rapid uptake and relatively slow clearance with a maximum SUV of 3.8 (370 MBq) and 3.3 (555 MBq) at 11–18 min after injection in the brain, and 4.9 (370 MBq) and 3.8 (555 MBq) at 8–14 min after injection in the kidneys (Figs. 2 and 3).

**Table 1:** Characteristics of study subjects.

	Injected dose (MBq)	
	370	555
Number	6	6
Age (y)	28.5 ± 6.7	32.7 ± 7.2
Height (cm)	168.7 ± 5.6	173.5 ± 4.2
Weight (kg)	65.8 ± 6.6	67.7 ± 5.2
Injected dose (MBq)	365.1 ± 8.6	564.2 ± 11.6
Injected mass (μg/kg)	0.291 ± 0.061	0.349 ± 0.069

Values are mean ± SD of six subjects per each group.

### Estimation of absorbed radiation doses in human

Table 2 shows the residence times of source organs. Relatively long residence times were observed in the liver, urinary bladder, kidneys, brain, and lungs (Table 2). The estimated absorbed radiation doses after the injection of either 370 MBq or 555 MBq of [^11^C]K-2 were averaged among the six subjects in each group and are listed in Table 3. The absorbed radiation dose of each organ was within the same range for individuals injected with 370 MBq or 555 MBq (Table 3). The urinary bladder showed the highest absorbed radiation doses, 31.2 ± 13.3 (370 MBq) and 25.9 ± 10.8 (555 MBq) μGy/MBq after the radiotracer injection, and the kidneys also showed higher absorbed radiation doses, 24.9 ± 3.2 (370 MBq) and 22.8 ± 4.9 (555 MBq) μGy/MBq after radiotracer injection, whereas the gallbladder wall had relatively low absorbed radiation doses, 6.7 ± 0.4 (370 MBq) and 6.7 ± 1.0 (555 MBq) μGy/MBq after radiotracer injection. These results indicate that [^11^C]K-2 was excreted mainly through the kidney and not through the liver-biliary tract. The liver also showed higher absorbed radiation doses than the other organs (22.1 ± 2.7 [370 MBq] and 20.9 ± 3.6 [555 MBq] μGy/MBq). Taking the overall absorbed radiation doses, recalculated to equivalent doses, the average effective dose per injected activity was estimated to be 5.2 ± 0.4 (370 MBq) and 5.0 ± 0.4 (555 MBq) μSv/MBq.

**Table 2:** Residence times of source organs determined by whole-body imaging with [^11^C]K-2 in male subjects.

370 MBq of [^11^C]K-2		555 MBq of [^11^C]K-2	
Source organ	Residence time (h)	Source organ	Residence time (h)
Brain	0.0246 ± 0.0034	Brain	0.0218 ± 0.0034
Thyroid	0.0001 ± 0.0000	Thyroid	0.0001 ± 0.0000
Lung	0.0238 ± 0.0049	Lung	0.0266 ± 0.0070
Heart	0.0028 ± 0.0010	Heart	0.0037 ± 0.0010
Spleen	0.0015 ± 0.0012	Spleen	0.0018 ± 0.0008
Liver	0.1188 ± 0.0148	Liver	0.1117 ± 0.0203
Stomach	0.0027 ± 0.0009	Stomach	0.0027 ± 0.0010
Kidney	0.0268 ± 0.0037	Kidney	0.0243 ± 0.0057
Pancreas	0.0012 ± 0.0006	Pancreas	0.0015 ± 0.0006
Gall bladder	0.0002 ± 0.0001	Gall bladder	0.0003 ± 0.0002
Small intestine	0.0114 ± 0.0036	Small intestine	0.0081 ± 0.0004
Urinary bladder	0.0431 ± 0.0194	Urinary bladder	0.0352 ± 0.0157
Red marrow	0.0155 ± 0.0011	Red marrow	0.0164 ± 0.0023
Remainder	0.2174 ± 0.0127	Remainder	0.2358 ± 0.0276

Values are mean ± SD of six subjects.

**Table 3:** Estimated absorbed radiation doses for different doses of [^11^C]K-2 in male subjects.

370 MBq of [^11^C]K-2		555 MBq of [^11^C]K-2	
Organ	Dose (μGy/MBq)	Organ	Dose (μGy/MBq)
Adrenals	6.6 ± 0.6	Adrenals	6.4 ± 0.8
Brain	5.7 ± 0.7	Brain	4.4 ± 1.9
Esophagus	2.9 ± 0.2	Esophagus	3.0 ± 0.1
Eyes	1.6 ± 0.1	Eyes	1.6 ± 0.2
Gallbladder wall	6.7 ± 0.4	Gallbladder wall	6.7 ± 1.0
Upper large intestine	2.8 ± 0.1	Upper large intestine	2.8 ± 0.1
Small intestine	6.8 ± 1.4	Small intestine	5.6 ± 0.2
Stomach wall	4.3 ± 0.6	Stomach wall	4.4 ± 0.7
Lower large intestine	3.1 ± 0.1	Lower large intestine	3.1 ± 0.1
Rectum	2.9 ± 0.5	Rectum	2.8 ± 0.4
Heart wall	3.9 ± 0.4	Heart wall	4.2 ± 0.4
Kidneys	24.9 ± 3.2	Kidneys	22.8 ± 4.9
Liver	22.1 ± 2.7	Liver	20.9 ± 3.6
Lungs	6.5 ± 1.1	Lungs	7.1 ± 1.4
Pancreas	5.0 ± 1.0	Pancreas	5.5 ± 1.1
Prostate	3.3 ± 0.6	Prostate	3.1 ± 0.5
Salivary glands	1.8 ± 0.1	Salivary glands	1.8 ± 0.2
Red marrow	3.6 ± 0.1	Red marrow	3.7 ± 0.2
Osteogenic cells	2.5 ± 0.1	Osteogenic cells	2.6 ± 0.1
Spleen	4.4 ± 2.2	Spleen	4.9 ± 1.6
Testes	1.7 ± 0.2	Testes	1.8 ± 0.2
Thymus	2.2 ± 0.1	Thymus	2.3 ± 0.1
Thyroid	2.1 ± 0.2	Thyroid	2.4 ± 0.1
Urinary bladder wall	31.2 ± 13.3	Urinary bladder wall	25.9 ± 10.8
Effective dose (μSv/MBq)	5.2 ± 0.4	Effective dose (μSv/MBq)	5.0 ± 0.4

Values are mean ± SD of six subjects.

**Figure 4:**
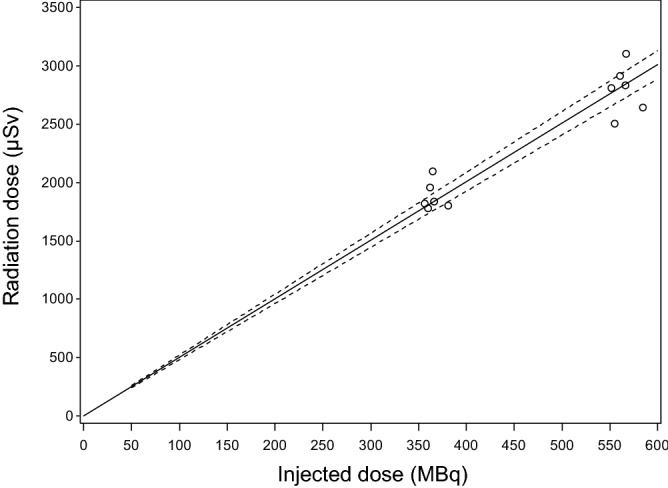
The dose-exposure relationship between injected dose and whole-body radiation dose after injection of [^11^C]K-2. Open circles indicate individual points obtained from each subject. Solid line indicates the estimated value and dashed lines indicate the edges of the 95% confidence interval.

To evaluate the relationship between injected dose and whole-body radiation dose, we analyzed these doses using fractional polynomial regression analysis with one degree of freedom, indicating that the dose-exposure relationship was linear after injection of [^11^C]K-2 (Fig. 4)^19^. This result was consistent with the observation that the average effective dose was almost the same between the 370 MBq-injected group and 555 MBq-injected group, because the whole-body radiation dose (μSv) was calculated as the product of the effective dose (μSv/MBq) and injected dose (MBq).

We also estimated absorbed radiation doses in female, based on the data of residence time obtained from male subjects. The urinary bladder showed the highest absorbed radiation doses, 37.3 ± 16.0 (370 MBq) and 30.9 ± 12.9 (555 MBq) μGy/MBq after the radiotracer injection, and the liver and kidneys also showed higher absorbed radiation doses in female (Table 4). Taking the overall absorbed radiation doses, recalculated to equivalent doses, the average effective dose per injected activity was estimated to be 6.5 ± 0.4 (370 MBq) and 6.3 ± 0.5 (555 MBq) μSv/MBq.

**Table 4:** Estimated absorbed radiation doses for different doses of [^11^C]K-2 in female subjects.

370 MBq of [^11^C]K-2		555 MBq of [^11^C]K-2	
Organ	Dose (μGy/MBq)	Organ	Dose (μGy/MBq)
Adrenals	6.9 ± 0.7	Adrenals	6.7 ± 0.8
Brain	6.4 ± 0.8	Brain	5.7 ± 0.8
Breasts	2.0 ± 0.1	Breasts	2.1 ± 0.1
Esophagus	3.6 ± 0.2	Esophagus	3.0 ± 0.2
Eyes	2.0 ± 0.1	Eyes	2.0 ± 0.1
Gallbladder wall	6.1 ± 0.3	Gallbladder wall	6.1 ± 0.8
Upper large intestine	3.2 ± 0.1	Upper large intestine	3.2 ± 0.1
Small intestine	8.3 ± 1.7	Small intestine	6.8 ± 0.2
Stomach wall	4.9 ± 0.6	Stomach wall	4.9 ± 0.7
Lower large intestine	3.4 ± 0.1	Lower large intestine	3.4 ± 0.1
Rectum	4.5 ± 1.1	Rectum	4.2 ± 0.8
Heart wall	4.2 ± 0.5	Heart wall	4.7 ± 0.5
Kidneys	28.0 ± 3.6	Kidneys	25.6 ± 5.5
Liver	27.8 ± 3.3	Liver	26.3 ± 4.5
Ovaries	8.0 ± 1.3	Ovaries	8.7 ± 1.7
Lungs	3.4 ± 0.5	Lungs	3.3 ± 0.4
Pancreas	6.8 ± 1.1	Pancreas	7.2 ± 1.3
Salivary glands	2.1 ± 0.1	Salivary glands	2.2 ± 0.1
Red marrow	4.4 ± 0.1	Red marrow	4.6 ± 0.2
Osteogenic cells	2.9 ± 0.1	Osteogenic cells	2.9 ± 0.1
Spleen	5.5 ± 2.5	Spleen	6.0 ± 1.8
Thymus	2.8 ± 0.1	Thymus	3.0 ± 0.1
Thyroid	2.3 ± 0.3	Thyroid	3.7 ± 0.4
Urinary bladder wall	37.3 ± 16.0	Urinary bladder wall	30.9 ± 12.9
Uterus	4.7 ± 1.1	Uterus	4.4 ± 0.8
Effective dose (μSv/MBq)	6.5 ± 0.4	Effective dose (μSv/MBq)	6.3 ± 0.5

Values are mean ± SD of six subjects.

During this clinical study, no adverse events related to the injection of [^11^C]K-2 were observed and no significant changes in vital signs (heart rate, blood pressure, respiratory rate, and saturation of percutaneous oxygen [SpO_2_]) during PET scan after the injection of [^11^C]K-2 were detected.

## Discussion

This study was the first clinical trial to directly evaluate the biodistribution and whole-body radiation dose measurements of [^11^C]K-2, the first radiotracer for AMPARs available in humans. The organ radiation exposure of [^11^C]K-2 was measured in 12 healthy subjects. We observed no adverse events related to this tracer. The highest uptake of [^11^C]K-2 was observed in the liver. The brain also showed relatively high uptake. Furthermore, biodistribution of source organs in mice, examined prior to human dosimetry, was also presented to compare the biodistribution between human and rodent. The urinary bladder and kidneys were critical organs for the excretion in human, whereas mice exhibited rapid reduction of the uptake of [^11^C]K-2 in the kidneys and sustained uptake in the liver. This result suggests that excretion route of [^11^C]K-2 were different among species; feces excretion is the major pathway in mice, while urinary excretion is dominant in human. It should be noted that residence time of mice urinary bladder was small compared with that of human due to exclusion of urine data from data set for calculating absorbed radiation dose in mice.

High absorbed radiation doses were observed in the bladder wall, kidney, and liver. The mean effective dose was 5.2 μSv/MBq in the 370 MBq-injected group and 5.0 μSv/MBq in the 555 MBq-injected group, which were comparable to other ^11^C-labeled tracers in the range of 3–16 μSv/MBq^20,21^. This low radiation burden allows obtaining serial PET scans in the same subject^22,23^. The mean effective doses were comparable between the two injected doses, indicating the linear dose-exposure relationship after injection of [^11^C]K-2. This result suggests that [^11^C]K-2 had no dose effect on the effective dose; therefore, there is no need for another human dosimetry trial to elucidate the effective dose of [^11^C]K-2 when an injected dose between 370 and 555 MBq is applied.

Here, we discuss the dose limit for [^11^C]K-2 referring to two guidelines, ICRP103 and Radioactive Drug Research Committee (RDRC). ICRP103 recommended that a whole-body radiation dose of 10 mSv is the limit for proof-of-concept research in healthy subjects^24^. Based on calculations using the mean effective dose, PET scans after [^11^C]K-2 intravenous injections resulted in the whole-body radiation dose of 1.92 mSv in the 370 MBq-injected group and 2.78 mSv in the 555 MBq-injected group, which are below 10 mSv, suggested by ICRP103. According to the guidelines of RDRC operating in the United States, the dose limit for adults is defined as follows: 30 mSv per single injection for the whole body, lens of the eyes, red marrow, and gonads; 50 mSv per year for the whole body, lens of the eyes, red marrow, and gonads; 50 mSv per single injection for all other organs; and 150 mSv per year for all other organs (FDA 21 CFR 361.1). As noted above, the urinary bladder wall was a critical organ and showed the highest absorbed radiation doses with 31.2 ± 13.3 μGy/MBq (370 MBq injection) and 25.9 ± 10.8 μGy/MBq (555 MBq injection). We can calculate the maximal injectable dose of [^11^C]K-2 for 1.60 GBq per single injection based on the following equation: the maximal injectable dose is calculated as the value of the dose limit for the urinary bladder wall divided by the absorbed radiation dose of the urinary bladder wall.

Since the radiation dose was obtained from healthy male subjects in this study, we next estimated the absorbed radiation doses and effective dose in female subjects. Based on the data obtained from male subjects collected in this study, the absorbed radiation doses in female were elucidated using the ICRP 89 Adult Female model in OLINDA 2.0, showing higher doses in most of source organs and effective dose estimates in comparison with male. The effective dose of female subjects was calculated as 6.48 μSv/MBq in 370 MBq-injected and 6.28 μSv/MBq in 555 MBq-injected individuals. The differences in effective dose between male and female depends on the type of radioligand; [^11^C]K-2 has a higher effective dose in women than in men. This could be due to the additional exposure of the breasts and uterus^25^. More importantly, the female phantom has smaller organ sizes such as brain, intestines, liver, urinary bladder, kidneys and this difference in female leads to higher dose deposition per organ compared to the male, leading to higher effective dose estimates. The whole-body radiation dose after [^11^C]K-2 intravenous injections is expected to be 2.40 mSv in the 370 MBq-injected group and 3.49 mSv in the 555 MBq-injected group, which are also below 10 mSv. However, the radiation sensitivity of women is reported to be higher so we need to consider carefully the benefit in the use of [^11^C]K-2 in women^26^.

We demonstrated that the effective dose of [^11^C]K-2 was as low as other [^11^C] PET tracers. The observed low effective dose of [^11^C]K-2 enables us to conduct repeated imaging to elucidate the biological basis and potential therapeutic targets of neuropsychiatric disorders.

## Conclusions

We evaluated the human whole-body biodistribution and radiation dosimetry of [^11^C]K-2, a radiotracer for AMPARs. The effective dose ranged from 5.0 to 5.2 μSv/MBq in male and 6.3 to 6.5 μSv/MBq in female. Thus, injections of 370 to 555 MBq (10 to 15 mCi) for PET studies using this radiotracer can be used safely in human subjects. [^11^C]K-2 also has a dosimetry profile that enables serial PET scanning in a single subject.

## Materials and methods

### Ethics statement

Animal studies were performed in strict accordance with the recommendations in the Guide for the Care and Use of Laboratory Animals of the National Institute of Radiological Sciences. The protocols were approved by the Committee on the Ethics of Animal Experiments of the National Institute of Radiological Sciences (approval number 07-1053-5). Clinical study was performed upon the approval from Yokohama City University Hospital Institutional Review Board and the Pharmaceuticals and Medical Devices Agency (PMDA) in accordance with the ministerial ordinance on standards for conducting clinical trials for medical devices (GCP ordinance) drawn up by the Japan Ministry of Health, Labour and Welfare as well as the Declaration of Helsinki. The clinical studies were also registered under UMIN000026357 (registration date 01/03/2017). All participants provided written informed consent after receiving detailed information about the study protocol.

### Mice dosimetry

Thirty six male ddY mice (8 weeks old, 35–40 g; Japan SLC) were used in the current study, with each colony containing four mice in SPF facility. Mice were kept monitored their health conditions and maintained on a 14:10 h light/dark cycle (full light at 0500 h and full darkness at 1900 h) with constant temperature and humidity at 22 ± 1 °C and 55 ± 5%, respectively. Food and water were provided ad libitum. Mice were restrained to plastic cylinder, followed by the bolus injection of [^11^C]K-2 (4.6–4.7 MBq/0.1 mL, 30–40 pmol) via the tail vein. At each experimental time point (1, 5, 15, 30, 60, and 90 min after the injection of [^11^C]K-2), organs were collected from four mice after cervical dislocation which were performed by well-trained individuals using the method recommended by the American Veterinary Medical Association Guidelines for Euthanasia of Animals: 2020 Edition. The whole brain, heart, liver, lung, spleen, testes, kidneys, pancreas, stomach (including contents), small intestine (including contents), large intestines (including contents), muscle, thigh bone, and blood samples were removed quickly. The radioactivity in these tissues was measured with an auto-gamma scintillation counter, Wizard 2480 (Perkin-Elmer, Waltham, MA, USA), and expressed as %ID/g. All radioactivity measurements were corrected for decay. To estimate the organ absorbed and effective doses, we extrapolated the data from mice to standard human using OLINDA 1.1 software (Vanderbilt University, Nashville, TN, USA). All procedures were confirmed to comply with ARRIVE guidelines.

### Participants

Thirty seven healthy male individuals were recruited and all subjects underwent medical screening performed by medical doctors confirming that they had not taken any medication at the time of screening and could keep absent from medication by the next day of [^11^C]K-2 PET examination, did not smoke, and had no history of medical illness, including hypertension, diabetes, organ failure, and neuropsychiatric disorders, on the basis of a medical interview, physical examination, blood examinations, and urinalysis prior to their participation in this study. Eighteen healthy male individuals passed screening and got registered in this study. Due to failures in synthesis of [^11^C]K-2, finally twelve healthy male subjects were divided into two groups, 370 and 555 MBq injection of [^11^C]K-2. During [^11^C]K-2 PET examination, the following vital signs were monitored in subjects: heart rate, blood pressure, respiratory rate, and saturation of percutaneous oxygen (SpO_2_). On the day the subjects underwent [^11^C]K-2 PET examination, they stayed one night in Yokohama City University Hospital to ensure there had been no serious adverse effects. The day after PET examination, the subjects were discharged from the hospital after receiving a medical check-up from a doctor. Subjects were required to revisit Yokohama City University Hospital to undergo a medical check to elucidate any adverse events related to this study.

### PET scans

[^11^C]K-2 was synthesized at Yokohama City University Hospital in accordance with the GMP ordinance and was certified by the Japanese Society of Nuclear Medicine. PET imaging was performed using a Canon Aquiduo scanner (Canon Medical Systems Corp., Tochigi, Japan), which provided an axial field-of-view (FOV) of 162 mm and 80 contiguous 2.0-mm thick slices. The PET device was calibrated daily with a ^68^Ge-^68^Ga source and cross-calibrated yearly to a dose calibrator using ^18^F activity contained in a 20-cm-diameter cylindrical phantom. A transmission computed tomography (CT) scan was performed for attenuation correction, then a 60-s intravenous injection of [^11^C]K-2 (370 MBq group: 365.1 ± 8.6 MBq, 555 MBq group: 564.1 ± 11.6 MBq) was administered. Each subject was scanned in 11 contiguous bed positions (head to knee) with an overlap of 10 frames, each of increasing duration (15 s–90 s for each bed position: 1f., 15 s × 11 bed positions; 2f., 15 s × 11 beds; 3f., 30 s × 11 beds; 4f., 30 s × 11 beds; 5f., 60 s × 11 beds; 6f., 60 s × 11 beds; 7f., 60 s × 11 beds; 8f., 90 s × 11 beds; 9f., 90 s × 11 beds; 10f., 90 s × 11 beds) for a total scan time of 120 min. There was a 7-s delay between the bed positions and a 30-s delay between frames for the movement of the bed^27^. Dynamic images were reconstructed with a filtered back projection method using a 128 matrix with a 4 × 4 × 2-mm voxel size and 7.0-mm Gaussian filter, corrected for scatter^28^.

### Image analysis

Following source organs were visually identified on PET images at the following time points after [11C]K-2 injection: brain, frame 4; heart, frame 1; liver, frame 6; kidney, frame 1; small intestine, frame 2; urinary bladder, frame 10. Thyroid, lung, spleen, stomach, pancreas, gall bladder and lumber spine (used to obtain the radioactivity of red marrow) were difficult to identify on PET images and were visually identified on CT images. Regions of interest (ROIs) on source organs were drawn on a slice-by-slice basis on CT images registered with the PET images and adjusted to visually circumscribe most of the activity within the respective source organ of the PET images. Images were analyzed using PMOD 3.709 software (PMOD Technologies, Zurich, Switzerland) to elucidate the time-activity curve of each organ.

### Calculation of residence time, absorbed radiation dose and effective dose

The calculation of residence time was based on a previously reported method^29^. The residence time in each source organ was calculated as the area under the curve (AUC) of the time-activity curve, which was created after removing decay correction and expressed as a percentage of injected activity after applying a recovery correction. The time-activity curves were created using time information of the bed position in which the source organ was primarily included when the organ was not confined to a single bed position. The AUC to the end of imaging was calculated using the trapezoidal method, and from the end to infinity was calculated by assuming that any further decline in radioactivity occurred only due to physical decay with no biological clearance. This assumption should not introduce any significant bias because the activity was very small at the last time point, which was approximately 120 min or almost six half-lives after injection. The residence time for all red marrow in the body was estimated from that in the lumbar vertebrae. To conservatively estimate the absorbed radiation doses, we assigned all radioactivity in the lumbar vertebrae to red marrow. Because the mass of red marrow in the lumbar vertebrae is 11.7% of the mass of all red marrow in the body (ICRP Publication 89), the residence time in red marrow in the entire body was calculated by multiplying that of the lumbar vertebrae by 100/11.7. The residence time of all source organs was summed and subtracted from the fixed theoretical value of T_1/2_/ln 2, which is 0.490 h for ^11^C.

The Absorbed radiation doses in male were calculated based on the Medical Internal Radiation Dose scheme by entering the residence times of each male subject for each source organ into OLINDA 2.0 software (Vanderbilt University, Nashville, TN, USA) for ICRP 89 Adult Male. Absorbed radiation doses in female were also calculated by entering residence time of each male subject into OLINDA 2.0 software for ICRP 89 Adult Female.

The effective doses were also calculated in OLINDA 2.0 based on the recommendation in ICRP Publication 103 using the absorbed radiation doses.

### Statistics

To evaluate the relationship between injected dose and radiation dose, we analyzed these values using fractional polynomial regression analysis. We used a linear regression with the fractional polynomial with one degree of freedom for *Z* with power *k* given by

$$ Y = \beta_{0} + \beta_{{1}} Z^{k} + \varepsilon $$

where *Z* denotes the *j*th injected dose and *Y* denotes the radiation dose.

Here, the power, *k*, was taken from a set {− 2, − 1, − 0.5, 0, 0.5, 1, 2, 3}, where *Z*^0^ denotes log(*Z*) and *ε* is a random error term. All values of *k* were examined and the model deviance was used to select the best-fitting model^19^. The best-fitting model was the linear model (*k* = 1), indicating that the dose-exposure relationship was a linear dose–response relationship for the injection of [^11^C]K-2.

## Supplementary information

Supplementary Information 1.
