# Supplementary material for: Biodistribution and radiation dosimetry of the positron emission tomography probe for AMPA receptor, [11C]K-2, in healthy human subjects
Source: Sci Rep. 2021 Jan 15;11:1598. doi: 10.1038/s41598-021-81002-3 (PMC7810729; doi:10.1038/s41598-021-81002-3)
Supplement: Supplementary file 1 — Supplementary Information 1. [file 41598_2021_81002_MOESM1_ESM.docx]

**Supplementary information**

**Supplemental Table 1.**  Serial uptake of [^11^C]K-2 in source organs of male ddY mice.

**Supplemental Table 2.** Residence time of source organs determined by biodistribution of [^11^C]K-2 in male ddY mice.

**Supplemental Table 3.** Estimation of absorbed radiation doses in human using residence time of source organs in ddY mice injected [^11^C]K-2.
